# 

**Published:** 1838

**Authors:** 


### II.—ANALYTICAL AND ORIGINAL.

### THE WESTERN JOURNAL.

*E Sylvis Nuncius.*

CINCINNATI, OHIO, APRIL 1, 1838.

### I.—To our Subscribers.

It is our intention, after the publication of this number of the Western Journal, to send it to none of our subscribers, out of the city, who do not pay in advance. In the midst of professional and professorial engagements, we find little time, and no fit disposition of mind, or business capacity, for superintending a periodical pnblication, on the credit system; especially, when it circulates through 15 or 18 states of the Union, and, of course, requires a large number of agents—both travelling and stationary. Such of our friends, therefore, as may desire to continue their patronage, will please to bear in mind, the necessity of remitting the subscription money, three dollars, for the 12th volume, before the first day of July next, when the first number of that volume will be ready for distribution. They, however, who may forget to do it, can make the remittance, after that time, and the number will be forwarded to them. According to our present practice, we shall receive, at par, the bank bills of the solvent banks of the states in which subscribers reside; but hope, that those who live out of Ohio, Kentucky, Indiana and Illinois, will, when they find it practicable, remit us the notes of some one of the banks of those states. The remittance will be at our risk. In all cases, the postage must be paid.

DAN DRAKE,
LANDON C. RIVES,
*Publishing Committee.*

### Art. XV.—To the Patrons of the Western Journal.

*To Delinquent Subscribers.*—The reason for advancing the subscription price of our Journal from three to four dollars, if payment be not made during the publication of the volume, is, that commissions have to be given to collectors. We greatly prefer to have the sum of three dollars, remitted by mail or otherwise, to collecting four; and are of opinion, that our subscribers ought to have the same preference. We have concluded, therefore, to extend the time within which they may remit three dollars, until they receive the present number; when we hope that *all* who are in arrears, will, respectively, enclose us that sum. Such as wish to continue patrons of the work, will please to recollect, that it will, hereafter, be sent only to those who remit their subscriptions *in advance.* When, therefore, they are transmitting subscription money for the 11th volume, it would be well to enclose a remittance of the same amount, for the 12th; the first number of which will be issued at the end of June.

*Our next volume.*—As the undersigned will be occupied throughout the spring, summer and autumn of the present year, in the composition of the work on the Elements of Pathology announced in this number, he will be under the necessity of withdrawing a portion of time and attention, from the Editorial duties of the Journal; leaving it in the hands of his able colleagues, professors Gross and Harrison; who will, he trusts, conduct it in such a manner as to give it new interest to every reader. He proposes, however, to insert in the July number one of the chapters of his contemplated first book of pathology, as a specimen of the work.

D.

### BIBLIOGRAPHICAL RECORD;

### OR, QUARTERLY LIST OF NEW PUBLICATIONS IN MEDICINE AND SURGERY, AND THEIR ASSOCIATE SCIENCES.*

AMERICAN.

1. Introductory Lecture to a Course of Surgery, delivered in the Chemical Hall of the Washington Medical College of Baltimore. By John W. Dunbar, M. D. Professor of Surgery, and Surgeon to the College Hospital.—Baltimore: 1837.

2. A Clinical Lecture on the Primary Treatment of Injuries, delivered at the New York Hospital. By Alexander Stevens, M. D. Surgeon to the New York Hospital and emeritus Professor of Clinical Surgery. New York: 1837.

3. Practical Elocution; designed for the promotion of health, the cure of Stammering, &c. By Andrew Comstock, M. D. Philadelphia: 1837.

4. Lecture Introductory to the course on Pathology and Practice of Medicine in the University of Virginia. By R. E. Griffith, M. D. Charlottesville: 1837.

5. Introductory Lecture to the course of the Institutes of Medicine, in the University of Pennsylvania. By S. Jackson, M. D. Philadelphia: 1837.

6. The Philadelphia Practice of Midwifery. By Charles D. Meigs, M. D. Member of the American Philosophical Society. 12mo. pp.370. Philadelphia: 1838.

7. An Elementary Treatise on Midwifery, or Principles of Tokology and Embryology. By Alf. A. L. M. Velpeau, M. D. &c. Translated from the French by Ch. D. Meigs, M. D. Second Edition. 8vo. Philadelphia: 1838.

BRITISH.

1. Memoirs on the Nerves. By Marshall Hall, M. D. F. R. S. L. & E. 4to. With Plates. London: 1837.

2. On the Nature and Treatment of Diseases of the Heart; with some new views on the Physiology of the Circulation. By James Wardrop, M. D. 8vo. pp. 126. Part 1. Four Plates. London: 1837.

3. The Physician's Vade-Mecum; or Manual of the Principles and Practice of Physic. By Robert Hooper, M. D. New Edition, considerably Enlarged and Improved. By Michael Ryan M. D. 8vo. pp. 417. London: 1837.

4. An Experimental Essay on the Relative Physiological and Medicinal Properties of Iodine and its compounds. By Charles Cogswell, M. D, 8vo. pp. 179. Edinburgh: 1837.

* We would respectfully suggest to the authors of new works, the propriety of forwarding us copies, as soon after their publication as practicable. By so doing, they will always secure for them an early notice in our record. As things are now conducted, many of the eastern books never reach us until they are six or twelve months old.

5. First Principles of Medicine. By Archibald Billing, M. D. Second Edition. 8vo. pp. 130. London: 1837.

6. An Essay on Pyrexia, or Symtomatic Fever, as Illustrative of the Nature of Fever in General. By H. Clutterbuck, M. D. 8vo. pp. 136. London: 1837.

7. A Medico-Legal Treatise on Homicide by External Violence; with an account of the Circumstances which modify the Medico-Legal characters of Injuries and Exculpatory Pleas. By Alexander Watson, Esqr. Fellow of the Royal College of Surgeons. 8vo. pp. 355. Edinburgh: 1837.

8. On a New Mode of Treatment Employed in the Cure of Various Forms of Ulcer and Granulating Wounds. By F. C. S. Key, F. R. S. 8vo. pp. 85. London: 1837.

9. The Philosophy of the Eye; being a Familiar Exposition of its Mechanism and of the Phenomenon of Vision, with a view to the Evidence of Design. By John Walker, M. D. Lecturer on the Eye in the Manchester School of Medicine. With numerous Illustrations. 8vo. pp. 300. London: 1837.

10. Observation on the Topography, Climate, and Prevalent Diseases of the Island of Jersey; the Result of Meteorological Observations and General Practice during thirteen years. By G. S. Hooper, M. D. 8vo. pp. 199. London. 1837.

11. A Treatise on the Influenza of 1837; containing an analysis of 100 cases observed at Birmingham. By Payton Blakiston, M. A. 8vo. pp. 60. London: 1837.

12. An Exposition of the Signs and Symptoms of Pregnancy, the Period of Human Gestation, and the Signs of Delivery. By W. F. Montgomery, A. M. M. D. 8vo. London: 1837.

13. An Essay on the Classification of the Insane. By M. Allen, M. D. 8vo. pp. 208, with an appendix. London: 1837.

14. The Theory of Repulsion Examined in a series of Experiments. By Charles Hales, M. D. 8vo. pp. 22. London: 1837.

15. A Manual of Percussion and Anscultation. By Dr. Spillan. 8vo. London: 1837.

16. The Cyclopædia of Anatomy and Physiology. Edited by R. B. Todd, M. D. Part XI. London: 1837.

17. The Nature and Treatment of Diseases of the Ear. By Dr. W. Kramer. Translated from the German, with the latest improvements of the author. By Q. R. Bennett, M. D. &c. &c. 8vo. London: 1837.

18. Inaugural Dessertation on the presence of air in the organs of circulation. By Dr. J. R. Cormack. 8vo. Edinburgh: 1837.

19. Inaugural Dissertation on the Physiology and Pathology of the Brain. By Dr. J. H. Bennett. 8vo. Edinburgh: 1837.

20. Lectures Illustrative of certain Local Nervous affections. By Sir Benjamin C. Brodie, Bart. F. R. S. 8vo. London: 1837.

21. The Study of Medicine. By John Mason Good, M. D. F. R. S. Improved from the author's M. S. and by reference to the latest advances in Physiology, Pathology and Practice. By Samuel Cooper, Professor of Surgery in the University of London, &c. &c. 4th Edition. 4 thick 8vo. volumes. London: 1837.

### INDEX TO VOLUME XI.

A

Abdomen, binding of 125
Abscess, hepatic 369
" Psoas 18
Acclimation, 152
Alibert, his death 648
Alterants, 515
Amaurosis, 29
American Cyclopædia, 155
" Medical Schools, 571
Amputation of leg, 17
" of arm, 346
Anatomy, special 187
" morbid of liver 43
" of typhus 311
Anatomical School, 145
Animal Magnetism, 407
Anus diseases of 385

B

Bandage, permanent 463
Barton's Address, 152
Bandage for Prolapsus Uteri, 663
Belladonna in Ileus, 637
Bell's Library, 156
Bibliograph. Record, 169,503,676
Blindness, parturient 189
Bloodletting, 71
Body, pins taken from 300
Brain of the Negro, 181
" Ciliary motions, 462
Brandreth's Pills, 327
British Association, 499
" Journals. 500, 663
Bronchial Tubes, 460
Bushe on Rectum, 385

C

Carrall's Cases, 546
Carotid Artery tied, 105
" compressed, 623
Cartwright on Lobelia, 460
Catarrh of Bladder, 464
Cataract, 304, 349
Case-book extracts, 189
Chemistry of Digestion, 315
Cincinnati College, 173, 642
" Med Dep. 168,335,665
" Med. Society, 156
" Hospital, 9, 177, 345
Chlorine in Scarlatina, 123
Chorea, 640
Clinical Introductory, 34
Cream Tartar, poisoning from 623
College of Phys. and Surgs. 163
Cowle's Case, 192
Contagious Typhus, 313
Convention, Medical of Ohio, 337
507, 643
Compensation, 651
Cow Pox, 342
Crania, American 631
Cautery, actual 603
Creosote, 635

D

Death from fruit, 562
" from ulceration, 376
De Abortu, 332
Debate on Influenza, 445
Diseases of Larynx, 579
" of Uterus, 92
Diarrhœa, fatal 181

Diaphragm, height of 315
Dieffenbach on Pessaries, 302
Digestive Organs, 315
Delirium Tremens, 178
Drake's Elements, 643
Drowning, morbid appearance, 316
Dunglison's Library, 156
" Therapeutics, 199
Dropsy, after scarlet fever, 123

E

Eberle, his death, 650
Effects of Tannin, 192
Elm Bark in Surgery, 371
Endermic Medication, 379
Enlargement of Spleen, 454
Eye, Diseases of 225
" Morbid Sensibility 30
Epidemic Influenza, 441
Experiments on Arteries, 273
" of Sir A. Cooper, 105

F

Fees, doctor's 501
Factory System, 182
Fistula Lachrymalis, 353
Fever, Bilious, 182
" Intermittent, 355
" Typhoid, 187
Fœtal Monster, 340
French Hospitals, 161
Functions of the 5th, 6th & 7th pairs of Nerves, 189
Functions of Medula Oblongata *ib.*
" Spinalis, *ib.*
" Excito-Mot. Nerves *ib.*
Fungus Hœmatodes, 345

G

Graduates, 138
Galvanism, 325
Gerhard on Typhus, 489
Germany, discoveries, 319
General Therapeutics, 199
Graham's Journal, 320
Gross, his promised work, 499
Gossip, editorial, 497

H

Hamilton's Address, 145
Heart, Pulsations of 320
Heart, Sounds and Motions, 314
" Medicines on 88
Homœpathy, 88
Humerus, luxations of 124
Hydrocele, 301
Hydriodic Acid, 116
Hydriodate of Potash, 127

I

Indications of cure 33
Iodide of potassium 120
" of starch 122
Iodine, its action 116
" use in hydrocele 301
Indigo, its application 193
" operation 194
Influenza, debate on 441
" in Belgium 449
" Copenhagen 452
" France 441
Ives, his death 650
Jaundice 195

L

Liver, morbid anatomy of 43
Library of Cincinnati College 498
" Lane Seminary 166
Louisville Medical Institute 163

M

Medicine in Italy 317
Medical Examiner 644
" Societies in Ky. 339
" Associ'n of Ham. co. 165
" Department of Transylvania 150—162
" School of Fairfield 144
" Proceedings in Conn. 141
" Investigation, philosophy of, 156
" Profession 473
M'Intosh, his death 648
Milk sickness 322
Mesmer 409
Mount's Operation 341
Muscular fibre, elementary structure of 187

N

Neuralgia, miasmatic 190
" Intermittent 628
Nitrate of Silver 126—481—634

O

Odontalgia 555
Onychia Maligna 125
Obituary Notices 502
Opthalmia, cure of 303
Opium, poisonous effects 483
Orang-Outang's brain 181
Ova, first changes of 642
Ovarian Dropsy 190

P

Paralysis, treatment of 603
Parker's letter from Paris 160
Procpectus of this Journal 159
Pneumonia, of old people 306
Perforation of Stomach 485
Pessaries 465
Plug, use of 466
Pneumo-gastric tied 105
Polypi in ear 625
Physick's death 502
Pupil, artificial 26
" closed 23

Q

Quinine in large doses 321—357

R

Respiration, artificial 483
Researches, clinical 191
Report on Med. Col. of Ohio 670

S

Scalding, by steam 10
Sedative poisons 606
Statistics of hospitals 475
Saliva, indications of 179
Stomach, softening of 485
Septum narium malformed 630
Style, gentility of 499
Stricture in Urethra 125
Spleen, diseases of 454

T

Tendo Achilles, section of 470
Tracheotomy 482

U

Ulcers of legs 652
Uterus, diseases of 92—249
" laceration of 466

V

Vagina, lacerated 466
Vaginal obliteration 546
Ventricles, safety-valves of 181
Vermiform appendix, ulcer of 376
Voice, researches on 180

W

Western Journal 159

Women, Gonorrhœa in 381

